# Individualising Guselkumab Maintenance in Psoriatic Disease: Clinical Profiles Associated with Dosing-Interval Modification in a Multicentre Cohort

**DOI:** 10.3390/jcm15218567

**Published:** 2026-11-03

**Authors:** Edoardo Mortato, Marina Talamonti, Annunziata Raimondo, Viviana Lora, Matteo Megna, Cristina Mugheddu, Gabriele Biondi, Anna Balato, Giacomo Caldarola, Lorenzo Marcelli, Serena Lembo, Dario Graceffa, Luca Potestio, Laura Atzori, Maria Antonia Montesu, Eugenia Veronica Di Brizzi, Eleonora De Luca, Lucia Bufano, Luca Bianchi, Marco Galluzzo

**Affiliations:** 1 Department of Systems Medicine, University of Rome “Tor Vergata”, 00133 Rome, Italy; edoardo.mortato@gmail.com (E.M.); lmarcelli96@gmail.com (L.M.); luca.bianchi@uniroma2.it (L.B.); 2 Dermatology Unit, Azienda Ospedaliera Universitaria Policlinico “Tor Vergata”, 00133 Rome, Italy; marina.talamonti@ptvonline.it; 3 Department of Medicine Surgery and Dentistry, ‘Scuola Medica Salernitana’, University of Salerno, 84084 Salerno, Italy; araimondo@unisa.it (A.R.); slembo@unisa.it (S.L.); lbufano@unisa.it (L.B.); 4 Clinical Dermatology Unit, San Gallicano Dermatological Institute IRCCS, 00144 Rome, Italy; viviana_lora@yahoo.it (V.L.); dario.graceffa@ifo.it (D.G.); 5 Section of Dermatology, Department of Clinical Medicine and Surgery, University of Naples Federico II, 80131 Naples, Italy; mat24@libero.it (M.M.); potestioluca@gmail.com (L.P.); 6 Section of Dermatology, Department of Medical Sciences and Public Health, University of Cagliari, 09124 Cagliari, Italy; cristinamugheddu@tiscali.it (C.M.); bambule246@icloud.com (L.A.); 7 Dermatology Unit, Department of Medicine, Surgery and Pharmacy, University of Sassari, 07100 Sassari, Italy; babilon22@hotmail.com (G.B.); mmontesu@uniss.it (M.A.M.); 8 Dermatology Unit, University of Campania Luigi Vanvitelli, 801380 Naples, Italy; anna.balato@unicampania.it (A.B.); eugeniaveronica.dibrizzi@gmail.com (E.V.D.B.); 9 Dermatologia, Dipartimento Universitario di Medicina e Chirurgia Traslazionale, Università Cattolica del Sacro Cuore, 00168 Rome, Italy; giacomo.caldarola@unicatt.it (G.C.); deluca.eleonora94@gmail.com (E.D.L.); 10 Dermatologia, Dipartimento di Scienze Mediche e Chirurgiche, Fondazione Policlinico Universitario A. Gemelli IRCCS, 00168 Rome, Italy

**Keywords:** guselkumab, psoriasis, deep cutaneous response, interval adaptation, treatment trajectories, biologic dose reduction

## Abstract

**Background/Objectives**: Individualising guselkumab maintenance is increasingly considered in clinical practice, but the characteristics associated with clinicians’ decisions to modify dosing intervals remain poorly defined. We investigated the clinical profiles of patients undergoing interval extension or reduction and assessed drug survival after interval reduction in patients with psoriatic arthritis (PsA). **Methods**: This retrospective study included patients receiving guselkumab for psoriasis across eight Italian centres. Patients undergoing interval extension beyond every eight weeks (q8w; *n* = 39) were compared with those without interval extension (*n* = 951). Separately, patients with PsA undergoing interval reduction from q8w to every four weeks (q4w; *n* = 39) were compared with patients with PsA maintained on q8w (*n* = 184). Between-group comparisons were exploratory and unadjusted. Post-interval reduction drug survival was estimated using the Kaplan–Meier method. **Results**: Patients undergoing extension had less frequent palmoplantar involvement (2.6% versus 14.7%; *p* = 0.033), nail involvement (0% versus 13.6%; *p* = 0.014), and PsA (7.7% versus 23.1%; *p* = 0.023), and more frequent previous anti-IL-12/23 treatment (35.9% versus 18.2%; *p* = 0.006). Within the PsA population, interval reduction was associated with younger age (56.9 versus 62.8 years; *p* = 0.023) and obesity (41.0% versus 23.4%; *p* = 0.023). Five patients subsequently returned to standard q8w dosing following clinical improvement. All 39 patients undergoing interval reduction were included in the survival analysis, with six guselkumab discontinuations. Estimated drug survival was 97.1% at 52 weeks (95% CI 81.4–99.6%) and 84.2% at 104 weeks (95% CI 62.7–93.9%). **Conclusions**: Guselkumab interval modification was associated with distinct clinical profiles, suggesting that disease phenotype, joint involvement, and treatment history contribute to individualised maintenance decisions. These findings describe clinical selection in routine practice and support prospective evaluation of patient selection criteria and long-term outcomes.

## 1. Introduction

Guselkumab, an interleukin (IL)-23p19 inhibitor, is approved for plaque psoriasis and psoriatic arthritis (PsA). Standard maintenance treatment for psoriasis is 100 mg every eight weeks (q8w); European product information also permits every-four-week (q4w) dosing in selected patients with PsA at high risk of joint damage [1]. Trials in PsA have demonstrated efficacy of both schedules, providing a therapeutic framework for individualised maintenance [2,3,4].

Durable disease control has stimulated interest in adjusting treatment exposure. Dose-ranging and withdrawal–retreatment studies support the possibility that clinical benefit can persist beyond a scheduled injection, although this does not establish an appropriate maintenance interval for an individual patient [5,6,7]. Observational studies have described extended and disease-activity-guided regimens in selected patients with psoriasis [8,9,10,11,12]. The practical question is how clinicians select patients for these changes within the broader treated population.

We examined two clinically distinct treatment decisions in an eight-centre cohort of patients receiving guselkumab for psoriasis. Patients undergoing interval extension beyond q8w were compared with those maintained on standard q8w dosing. Separately, among patients with concomitant PsA, those undergoing interval reduction from q8w to q4w were compared with those maintained on standard q8w dosing. The primary aim was to characterise the clinical profiles associated with these treatment decisions. A secondary aim was to estimate drug survival after interval reduction.

## 2. Materials and Methods

### 2.1. Study Design and Comparison Groups

This retrospective observational study was conducted in eight Italian dermatology centres.

The study period extended from July 2025 to 31 March 2026. The source cohort included consecutive patients with plaque psoriasis, with or without concomitant PsA, who were receiving guselkumab at the participating centres during this period, including patients who had initiated guselkumab before July 2025 and remained on treatment at study entry. Patients were followed retrospectively through available clinical records until their last assessment or the administrative database cutoff of 31 March 2026.

**Figure 1:**
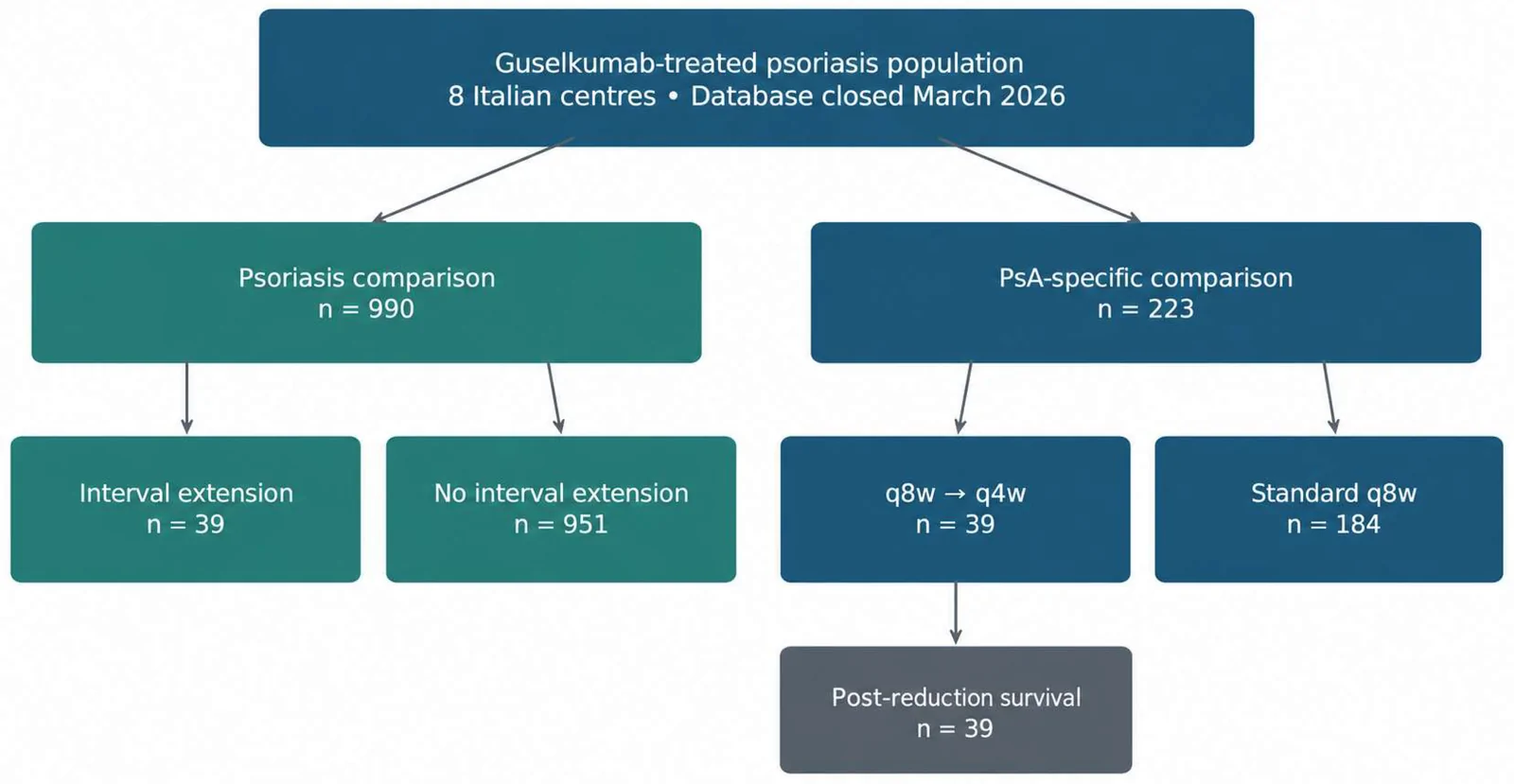
Study comparisons and the post-reduction survival population. The psoriasis analysis compared interval extension with no interval extension. The PsA-specific analysis compared interval reduction from q8w to q4w with continued standard q8w dosing. All 39 patients undergoing interval reduction were included in the post-reduction survival analysis. PsA, psoriatic arthritis; q4w/q8w, every four/eight weeks.

Demographic, clinical and treatment information was obtained from routine clinical records. Interval modifications were determined by treating clinicians during usual care, without random allocation or a study-mandated dosing algorithm. Interval reduction from q8w to q4w was undertaken in agreement with the treating rheumatologist in patients with persistent or inadequately controlled musculoskeletal manifestations. The specific clinical domain prompting interval reduction (e.g., peripheral joint activity, enthesitis or dactylitis) was not systematically recorded in the retrospective database and could therefore not be analysed separately. The psoriasis comparison included 990 patients: 39 underwent interval extension beyond every eight weeks (q8w), and 951 did not undergo extension. The PsA-specific comparison included 223 patients: 39 underwent interval reduction from q8w to every four weeks (q4w), whereas 184 remained on standard q8w dosing. The two comparisons addressed separate clinical questions and were not analysed as a direct comparison of extension against reduction (Figure 1).

Interval extension represented off-label maintenance treatment for psoriasis. Interval reduction increased administration frequency without reducing the dose per injection. Its regulatory context differs from extension: European product information permits q4w guselkumab in patients with PsA considered at high risk of joint damage. The q4w group was therefore not classified collectively as receiving off-label treatment.

### 2.2. Variables and Outcomes

Characteristics included age, sex, smoking, obesity, body mass index (BMI), involvement of the scalp, genital, palmoplantar and nail regions, comorbidities, PsA and previous biologic treatment. Guselkumab-related variables included age at initiation, psoriasis duration, baseline Psoriasis Area and Severity Index (PASI), time to interval modification, PASI at modification, and duration of observation.

The principal outcomes were between-group differences in these characteristics.

For patients undergoing interval reduction, drug survival was defined as the time from interval reduction to permanent guselkumab discontinuation for any reason. Return to the standard q8w regimen while continuing guselkumab was not considered a discontinuation event. Loss to follow-up was treated as a censoring event.

### 2.3. Statistical Analysis

Continuous variables were summarised as mean (standard deviation, SD). Categorical variables were expressed as *n*/N (%). Comparisons used Welch’s independent-samples *t*-test for continuous variables and Pearson’s chi-square test without continuity correction for categorical variables; Fisher’s exact test was used when any expected cell frequency was below five. All tests were two-sided. Comparisons were exploratory, without adjustment for multiple testing; *p* < 0.05 identified nominal statistical significance. No multivariable prediction model or causal treatment-effect analysis was fitted.

Drug survival after interval reduction was estimated using the Kaplan–Meier method, with 95% confidence intervals (CIs) calculated using Greenwood variance and a log–log transformation. Time was measured from interval reduction to permanent guselkumab discontinuation for any reason. Patients without documented discontinuation were censored at their last recorded assessment or the administrative cutoff of 31 March 2026, whichever occurred first. Loss to follow-up was censored at the last documented assessment. Subsequent return to standard q8w dosing did not constitute an event, as the analysis assessed persistence with guselkumab irrespective of dosing frequency. All 39 patients undergoing interval reduction were included.

No missing dates were imputed. Statistical analyses were performed using jamovi version 28.8.

## 3. Results

### 3.1. Clinical Profile Associated with Interval Extension

Thirty-nine patients underwent interval extension, representing 3.9% of the 990-patient comparison cohort. All extended guselkumab administration from every eight weeks (q8w) to every 12 weeks (q12w). Compared with patients with no interval extension, those undergoing extension had less frequent palmoplantar involvement (1/39, 2.6%, versus 140/951, 14.7%; *p* = 0.033), nail involvement (0/39 versus 129/951, 13.6%; *p* = 0.014) and PsA (3/39, 7.7%, versus 220/951, 23.1%; *p* = 0.023). Previous anti-IL-12/23 treatment was more frequent in the extension group (14/39, 35.9%, versus 173/951, 18.2%; *p* = 0.006) (Table 1).

Age, BMI, baseline PASI and overall previous biologic exposure did not differ significantly between groups. Cardiovascular disease was numerically less frequent among patients undergoing extension (5.1% versus 16.4%), although the difference did not reach nominal statistical significance (*p* = 0.060).

Interval extension occurred a mean of 96.1 weeks after guselkumab initiation. Skin disease activity was low at modification, with a mean PASI of 0.3 and a median of 0 (IQR 0–0). Mean observation duration was longer in the extension group than in patients maintained on standard dosing (212.5 versus 155.5 weeks; *p* < 0.001).

During follow-up, two patients discontinued guselkumab because of lower-limb arthralgia, and one patient was lost to follow-up. No patient required a return to standard q8w dosing during documented follow-up.

### 3.2. Clinical Profile Associated with Interval Reduction in PsA

Among the 223 patients with PsA, 39 (17.5%) underwent interval reduction from q8w to q4w. These patients were younger than those maintained on standard q8w dosing, both at the recorded age assessment (56.9 versus 62.8 years; *p* = 0.023) and at guselkumab initiation (53.0 versus 59.0 years; *p* = 0.020). Obesity was more frequent in the interval reduction group (16/39, 41.0%, versus 43/184, 23.4%; *p* = 0.023). No other between-group differences reached nominal statistical significance (Table 2). Mean baseline PASI was 12.7 versus 14.6 (*p* = 0.113), and mean observation duration was 178.6 versus 158.3 weeks (*p* = 0.221). Interval reduction occurred at a mean of 95.6 weeks after guselkumab initiation. Skin disease activity was low at modification, with a mean PASI of 0.9.

Following clinical improvement, five patients returned from q4w to the standard q8w regimen in agreement with their treating rheumatologist while continuing guselkumab. This return to q8w was considered a change in dosing interval and not treatment discontinuation.

### 3.3. Drug Survival After Interval Reduction

**Figure 2:**
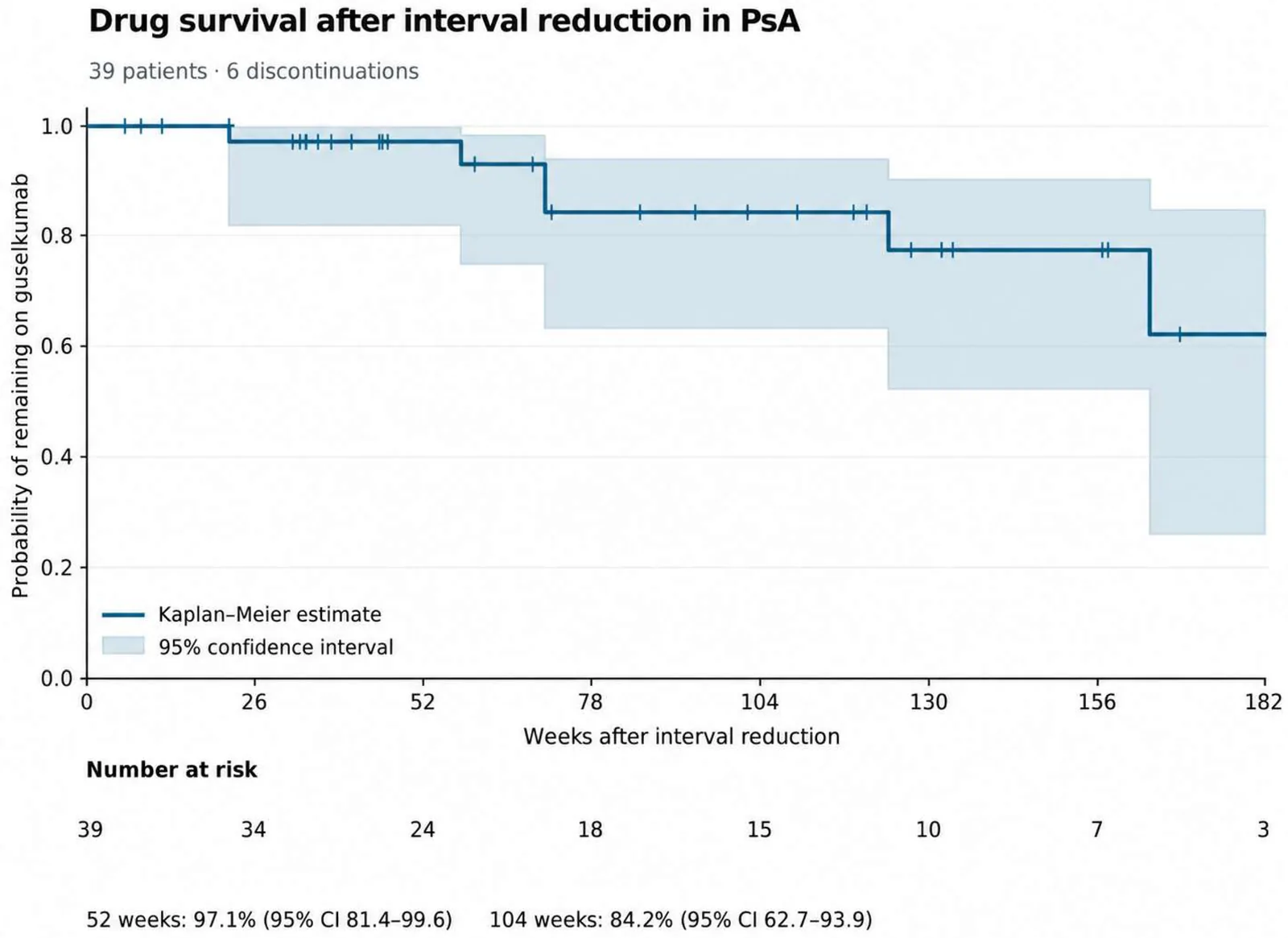
Kaplan-Meier drug survival after guselkumab interval reduction in patients with psoriatic arthritis. The analysis included 39 patients and six permanent treatment discontinuations. Time zero represents reduction from q8w to q4w dosing. Shading indicates 95% confidence intervals calculated using Greenwood variance and a log–log transformation; crosses indicate censored observations. Numbers at risk are displayed below the plot. Loss to follow-up was censored at the last documented assessment, and subsequent return to q8w dosing did not constitute discontinuation. The display is restricted to 182 weeks and includes all six discontinuation events; estimates in the later portion of the curve are based on few patients. PsA, psoriatic arthritis; q4w, every four weeks; q8w, every eight weeks.

All 39 patients undergoing interval reduction were included in the survival analysis, contributing 68.7 patient-years of observation. Median observed follow-up after reduction was 71.0 weeks (IQR 37.0–129.9). Six patients discontinued guselkumab: three because of loss of efficacy, including two with explicitly documented articular worsening, and three following the detection of pancreatic nodules, pulmonary lesions, and gastritis, respectively. These three medical events were considered unrelated to guselkumab by the treating clinicians. One additional patient was lost to follow-up and was censored at the last recorded assessment, 32 weeks after interval reduction. Estimated guselkumab drug survival was 97.1% at 52 weeks (95% CI 81.4–99.6%) and 84.2% at 104 weeks (95% CI 62.7–93.9%), with 24 and 15 patients remaining at risk, respectively. At 156 weeks, estimated survival was 77.2% (95% CI 51.7–90.3%), with seven patients at risk (Figure 2). Median drug survival was not reached. Patients who subsequently returned to q8w dosing continued to contribute follow-up while remaining on guselkumab.

## 4. Discussion

This study examines who undergoes guselkumab interval modification in clinical practice. The two comparisons suggest that clinicians’ maintenance decisions are associated with different patient profiles. Extension was less frequent in patients with palmoplantar or nail involvement and in those with PsA, whereas reduction within the PsA population was associated with younger age and obesity. The contribution of these analyses is to place modified-regimen patients within their relevant source populations, showing the clinical context in which clinicians elect to change dosing when no validated patient-selection algorithm is available.

The extension findings are compatible with cautious selection when disease involves anatomical sites or domains that may be insufficiently represented by an overall skin score. The absence of nail involvement and the low frequency of palmoplantar involvement in the extension group suggest that the distribution of disease may influence clinicians’ confidence in spacing treatment. The lower prevalence of PsA similarly points towards consideration of disease beyond the skin. These are plausible interpretations of observed associations: the study did not directly record the weight clinicians assigned to each characteristic or establish that these features independently determined the decision.

Previous anti-IL-12/23 therapy was more common among patients undergoing extension. These findings warrant attention but do not establish that previous exposure facilitates successful interval spacing. It could reflect treatment history, duration of established disease control, centre-specific prescribing practices or other unmeasured selection factors. The individual previous-treatment categories used the entire comparison group as their denominator; the finding should therefore not be interpreted as a comparison restricted to biologic-experienced patients. Longer observation in the extension group also provides more opportunity for a patient to reach a point at which spacing is considered.

The greater frequency of previous anti-IL-12/23 treatment also connects with reports of guselkumab interval optimisation after switching from ustekinumab. Other observational studies have described individualised or on-demand treatment and dose spacing with guselkumab or other IL-23 inhibitors [9,10,11,12]. These studies support the feasibility of selected maintenance strategies, but they do not establish that the characteristics associated with spacing in our cohort predict its success. BeNeBio adds randomised evidence for disease-activity-guided reduction across IL-17 and IL-23 inhibitors; its mixed-drug population does not provide a guselkumab-specific selection rule [13].

The super-responder concept provides a useful context for interpreting clinical selection. Super responders are defined as patients achieving PASI 0 at week 20 and examined for their subsequent skin outcomes [14]. Using this definition, our comparison groups showed no significant difference in super-responder frequency: 71.8% versus 63.3% for extension versus no extension (*p* = 0.280), and 66.7% versus 66.8% for reduction versus standard dosing within PsA (*p* = 0.983). These exploratory observations suggest that early complete clearance alone does not fully account for later prescribing decisions. GUIDE used a stricter definition, requiring PASI 0 at both weeks 20 and 28, and demonstrated noninferiority of q16w versus q8w for maintaining PASI below 3 at week 68 in this selected population [15]. The trial addresses the feasibility of spacing in eligible responders; our comparisons add information on the broader clinical profiles associated with selection in routine care.

The recent Delphi consensus helps contextualise extension as an individualised decision. It recommends controlled disease for at least six months, shared decision-making, consideration of quality of life, and rheumatology input when PsA coexists. It also emphasises monitoring and returning to a previously effective schedule when control is lost. For IL-17 and IL-23 inhibitors, the consensus allows individualisation while acknowledging limited evidence [16]. The low frequency of PsA among patients undergoing extension is consistent with this cautious approach, although the present retrospective study cannot establish adherence to all consensus criteria.

The PsA comparison used patients with the same joint diagnosis who remained on q8w as its reference group. This makes the higher prevalence of obesity and younger age in the reduction group clinically relevant observations within PsA, rather than consequences of comparing patients with and without arthritis. Low PASI at interval reduction suggests that cutaneous response alone did not explain the dosing decision. However, the absence of standardised longitudinal joint-activity measures limits interpretation of the reasons for reduction. Obesity cannot be treated as an indication for q4w dosing, and the two age findings represent related measurements rather than independent selection factors.

Evidence for shortening should be interpreted separately. A retrospective guselkumab case series described q6w or q4w optimisation for inadequate cutaneous response, whereas our comparison concerns patients with PsA [17]. DISCOVER-1, DISCOVER-2 and SOLSTICE evaluated q4w and q8w schedules assigned at treatment initiation, with sustained efficacy also reported over longer follow-up [2,3,4,18]. Analyses of q8w treatment suggest that responses can persist between consecutive doses [19]. These findings do not directly establish the benefit of switching from q8w to q4w after inadequate joint control. The new information from our cohort is the profile of patients selected for this change and their subsequent treatment persistence, rather than proof of comparative articular efficacy.

Guselkumab retention after interval reduction was high during the first year, with estimated drug survival of 97.1% at 52 weeks and 84.2% at 104 weeks. Five patients subsequently returned to standard q8w dosing following clinical improvement and agreement with their treating rheumatologist, illustrating that interval reduction may be followed by reassessment of maintenance requirements. Nevertheless, drug persistence does not establish control of arthritis or superiority of q4w over q8w. The small number of discontinuations and declining numbers at risk resulted in increasingly wide confidence intervals, particularly beyond two years. These findings support prospective evaluation of treatment persistence alongside validated joint outcomes and predefined criteria for maintaining, reversing, or discontinuing interval reduction.

Pharmacokinetic and exposure–response analyses provide a rationale for considering individual variation in maintenance requirements, while tissue studies describe immunological changes during IL-23 blockade [20,21,22]. Such findings remain insufficient to identify who can safely extend treatment or who will benefit from shortening. EULAR recommendations allow cautious tapering in sustained PsA remission, without defining a guselkumab-specific interval [23]. In the context of disease modification, treatment persistence and low skin activity should not be equated with altered disease course: prospective assessment of sustained skin and joint remission, structural outcomes, and the consequences of reduced exposure is still required.

The study is limited by its retrospective design, clinician-driven treatment selection, small modified-regimen groups, and numerous unadjusted comparisons. Centre effects, disease activity at the decision visit, duration of sustained remission, patient preference, and quality-of-life measures were not adequately captured for adjusted analyses, and differences in observation time may have influenced opportunities for interval modification. In the PsA subgroup, standardised longitudinal measures of musculoskeletal disease activity were not consistently available, and the specific domain prompting interval reduction—such as peripheral joint activity, enthesitis, or dactylitis—was not systematically recorded. Therefore, the findings should be considered exploratory and hypothesis-generating rather than as evidence of independent predictors, comparative efficacy, or a validated dosing algorithm. Prospective studies should incorporate predefined criteria for interval modification, validated skin and joint disease-activity measures, and longitudinal outcomes to determine whether these clinical profiles can reliably inform individualised guselkumab maintenance.

## 5. Conclusions

In this exploratory cohort, guselkumab interval extension was associated with well-controlled skin disease, less frequent palmoplantar and nail involvement, and less concomitant PsA. Conversely, interval reduction to q4w was associated with younger age and obesity within the PsA population. These findings describe clinical characteristics associated with real-world treatment decisions and should not be interpreted as independent predictors or validated criteria for interval modification. Individualised dosing requires regular reassessment of skin and joint disease and, when PsA is present, close collaboration with the treating rheumatologist.

## Figures and Tables

**Table 1:** Demographic, clinical and treatment characteristics of patients with and without guselkumab interval extension.

Characteristic	Interval Extension	No Interval Extension	*p*-Value
Patients, *n*	39	951	
Male sex, *n*/N (%)	24/39 (61.5%)	566/951 (59.5%)	0.801
Age, years	56.3 (17.6)	56.6 (17.6)	0.883
Smokers, *n*/N (%)	18/39 (46.2%)	413/951 (43.4%)	0.736
Obesity, *n*/N (%)	8/39 (20.5%)	224/951 (23.6%)	0.660
BMI, kg/m^2^	26.6 (7.3)	27.5 (4.8)	0.163
Scalp involvement, *n*/N (%)	12/39 (30.8%)	380/951 (40.0%)	0.250
Genital involvement, *n*/N (%)	11/39 (28.2%)	192/951 (20.2%)	0.224
Palmoplantar involvement, *n*/N (%)	1/39 (2.6%)	140/951 (14.7%)	**0.033**
Nail involvement, *n*/N (%)	0/39 (0.0%)	129/951 (13.6%)	**0.014**
At least one comorbidity, *n*/N (%)	28/39 (71.8%)	626/951 (65.8%)	0.440
Cardiovascular disease, *n*/N (%)	2/39 (5.1%)	156/951 (16.4%)	0.060
Previous neoplasia, *n*/N (%)	2/39 (5.1%)	54/951 (5.7%)	1.000 ^f^
LTBI, *n*/N (%)	4/39 (10.3%)	68/951 (7.2%)	0.521 ^f^
Age at guselkumab initiation, years	52.0 (17.6)	53.0 (17.6)	0.564
Psoriasis duration at guselkumab initiation, years	26.4 (15.7)	23.6 (15.8)	0.288
PsA, *n*/N (%)	3/39 (7.7%)	220/951 (23.1%)	**0.023**
Bio-experienced, *n*/N (%)	22/39 (56.4%)	492/951 (51.7%)	0.567
At least 3 previous biologics, *n*/N (%)	4/39 (10.3%)	82/951 (8.6%)	0.769 ^f^
Last anti-TNF therapy, *n*/N (%)	2/39 (5.1%)	121/951 (12.7%)	0.216 ^f^
Last anti-IL-17 therapy, *n*/N (%)	5/39 (12.8%)	185/951 (19.5%)	0.303
Last anti-IL-12/23 therapy, *n*/N (%)	14/39 (35.9%)	173/951 (18.2%)	**0.006**
Last anti-IL-23 therapy, *n*/N (%)	1/39 (2.6%)	13/951 (1.4%)	0.432 ^f^
PASI at baseline	11.7 (8.6)	13.7 (7.6)	0.172
Time from initiation to index modification, weeks	96.1 (69.9)	-	-
PASI at modification	0.3 (1.0)	-	-
Last observation, weeks	212.5 (100.2)	155.5 (105.1)	**<0.001**

Data are *n*/N (%) or mean (SD). Pearson’s chi-square test without continuity correction was used for categorical comparisons; ^f^ denotes Fisher’s exact test when an expected frequency was <5. Continuous comparisons used Welch’s *t*-test. All tests were two-sided and unadjusted for multiple testing. Bold *p*-values are <0.05. -, not applicable. BMI, body mass index; IL, interleukin; LTBI, latent tuberculosis infection; PASI, Psoriasis Area and Severity Index; PsA, psoriatic arthritis; SD, standard deviation; TNF, tumour necrosis factor; q8w, every eight weeks.

**Table 2:** Demographic, clinical and treatment characteristics of patients with PsA with and without guselkumab dose-interval reduction.

Characteristic	Interval Reduction	No Interval Reduction	*p*-Value
Patients, *n*	39	184	
Male sex, *n*/N (%)	20/39 (51.3%)	100/184 (54.3%)	0.727
Age, years	56.9 (17.6)	62.8 (23.7)	**0.023**
Smokers, *n*/N (%)	16/39 (41.0%)	78/184 (42.4%)	0.875
Obesity, *n*/N (%)	16/39 (41.0%)	43/184 (23.4%)	**0.023**
BMI, kg/m^2^	30.2 (7.3)	28. (12.1)	0.215
Scalp involvement, *n*/N (%)	20/39 (51.3%)	70/184 (38.0%)	0.126
Genital involvement, *n*/N (%)	10/39 (25.6%)	32/184 (17.4%)	0.231
Palmoplantar involvement, *n*/N (%)	7/39 (17.9%)	34/184 (18.5%)	0.938
Nail involvement, *n*/N (%)	6/39 (15.4%)	48/184 (26.1%)	0.156
At least one comorbidity, *n*/N (%)	34/39 (87.2%)	138/184 (75.0%)	0.100
Cardiovascular disease, *n*/N (%)	3/39 (7.7%)	35/184 (19.0%)	0.087
Previous neoplasia, *n*/N (%)	4/39 (10.3%)	17/184 (9.2%)	0.769 ^f^
LTBI, *n*/N (%)	2/39 (5.1%)	17/184 (9.2%)	0.539 ^f^
Age at guselkumab initiation, years	53.0 (17.6)	59.0 (23.6)	**0.020**
Psoriasis duration at guselkumab initiation, years	27.9 (15.8)	27.3 (16.6)	0.820
Bio-experienced, *n*/N (%)	23/39 (59.0%)	121/184 (65.8%)	0.421
At least 3 previous biologics, *n*/N (%)	4/39 (10.3%)	29/184 (15.8%)	0.379
Last anti-TNF therapy, *n*/N (%)	6/39 (15.4%)	33/184 (17.9%)	0.703
Last anti-IL-17 therapy, *n*/N (%)	10/39 (25.6%)	42/184 (22.8%)	0.706
Last anti-IL-12/23 therapy, *n*/N (%)	7/39 (17.9%)	43/184 (23.4%)	0.461
Last anti-IL-23 therapy, *n*/N (%)	0/39 (0.0%)	3/184 (1.6%)	1.000 ^f^
PASI at baseline	12.7 (8.3)	14.6 (8.4)	0.113
Time from initiation to index modification, weeks	95.6 (81.9)	-	-
PASI at modification	0.9 (1.4)	-	-
Last observation, weeks	178.6 (99.8)	158.3 (97.7)	0.221

Data are *n*/N (%) or mean (SD). Pearson’s chi-square test without continuity correction was used for categorical comparisons; ^f^ denotes Fisher’s exact test when an expected frequency was <5. Continuous comparisons used Welch’s *t*-test. All tests were two-sided and unadjusted for multiple testing. Bold *p*-values are <0.05. -, not applicable. BMI, body mass index; IL, interleukin; LTBI, latent tuberculosis infection; PASI, Psoriasis Area and Severity Index; PsA, psoriatic arthritis; SD, standard deviation; TNF, tumour necrosis factor; q8w, every eight weeks.

## Data Availability

The data that support the findings of this study are available on request from the corresponding author, M.G.

## Abbreviations

The following abbreviations are used in this manuscript:

BMI	Body mass index
CI	Confidence interval
IL	Interleukin
IQR	Interquartile range
KM	Kaplan–Meier
PASI	Psoriasis Area and Severity Index
PsA	Psoriatic arthritis
q4w	Every 4 weeks
q8w	Every 8 weeks
q12w	Every 12 weeks
q16w	Every 16 weeks
SD	Standard deviation

## References

[1] European Medicines Agency (2026). Tremfya: EPAR—Product Information.

[2] Mease P.J., Rahman P., Gottlieb A.B., Kollmeier A.P., Hsia E.C., Xu X.L., Sheng S., Agarwal P., Zhou B., Zhuang Y. (2020). Guselkumab in biologic-naive patients with active psoriatic arthritis (DISCOVER-2): A double-blind, randomised, placebo-controlled phase 3 trial. Lancet.

[3] Deodhar A., Helliwell P.S., Boehncke W.H., Kollmeier A.P., Hsia E.C., A Subramanian R., Xu X.L., Sheng S., Agarwal P., Zhou B. (2020). Guselkumab in patients with active psoriatic arthritis who were biologic-naive or had previously received TNFalpha inhibitor treatment (DISCOVER-1): A double-blind, randomised, placebo-controlled phase 3 trial. Lancet.

[4] Ogdie A., Merola J.F., Mease P.J., Ritchlin C.T., Scher J.U., Lafferty K.P., Chan D., Chakravarty S.D., Langholff W., Wang Y. (2026). Efficacy and safety of guselkumab in participants with active psoriatic arthritis after inadequate response to one prior tumour necrosis factor inhibitor: Week-24 results of the phase 3, randomised, placebo-controlled SOLSTICE study. Arthritis Rheumatol..

[5] Gordon K.B., Duffin K.C., Bissonnette R., Prinz J.C., Wasfi Y., Li S., Shen Y.-K., Szapary P., Randazzo B., Reich K. (2015). A phase 2 trial of guselkumab versus adalimumab for plaque psoriasis. N. Engl. J. Med..

[6] Reich K., Armstrong A.W., Foley P., Song M., Wasfi Y., Randazzo B., Li S., Shen Y.-K., Gordon K.B. (2017). Efficacy and safety of guselkumab compared with adalimumab for psoriasis with randomized withdrawal and retreatment: Results from VOYAGE 2. J. Am. Acad. Dermatol..

[7] Gordon K.B., Armstrong A.W., Foley P., Song M., Shen Y.-K., Li S., Muñoz-Elías E.J., Branigan P., Liu X., Reich K. (2019). Guselkumab efficacy after withdrawal is associated with suppression of serum IL-23-regulated IL-17 and IL-22 in psoriasis: VOYAGE 2 study. J. Investig. Dermatol..

[8] Ruiz-Villaverde R., Chinchay F.V., Rodriguez-Fernandez-Freire L., Armario-Hita J.C., Pérez-Gil A., Galán-Gutiérrez M. (2022). Guselkumab dosing interval optimization in adult patients with moderate-to-severe psoriasis switching from ustekinumab. Dermatol. Ther..

[9] Herranz-Pinto P., Alonso-Pacheco M.L., Feltes-Ochoa R., Mayor-Ibarguren A., Servera-Negre G., Busto-Leis J.M., Gonzalez-Fernández M.A., Herrero-Ambrosio A. (2023). Real-world performance of a new strategy for off-label use of guselkumab in moderate to severe psoriasis: Super-responder patients as the epitome of efficacy and optimisation. Clin. Drug Investig..

[10] Mastorino L., Dapavo P., Ortoncelli M., Bongiovanni E., Liao Y., Leo F., Quaglino P., Ribero S. (2025). Dose modulation strategies in psoriatic patients: Real-life pilot comparison between risankizumab and guselkumab up to 12 months after dose spacing. Exp. Dermatol..

[11] Cammarata E., Airoldi C., Colombo J., Ucciero A., Mastorino L., Arese V., Burzi L., Castelli F., Chiarpenello M., Graziola F. (2025). Impact of disease activity-guided dose reduction on IL-17 and IL-23 inhibitors in psoriasis: A real-world assessment of efficacy, safety, and economic benefits. Dermatol. Pract. Concept..

[12] Mastorino L., Dapavo P., Ortoncelli M., Bongiovanni E., Quaglino P., Ribero S. (2025). IL-17 and IL-23 inhibitors dose spacing in adult psoriatic patients: A real-world pilot study. J. Dtsch. Dermatol. Ges..

[13] van den Reek J.M.P.A., van Riel C.A.M., Soenen R., Eylenbosch A., Schots L., van der Schoot L.S., Grine L., Hannink G., Willaert F., Hillary T. (2026). Dose reduction of IL17 and IL23 inhibitors in psoriasis (BeNeBio study): An international, pragmatic, multicentre, randomised, controlled, non-inferiority trial. Lancet Reg. Health Eur..

[14] Mortato E., Talamonti M., Marcelli L., Megna M., Raimondo A., Caldarola G., Bernardini N., Balato A., Campanati A., Esposito M. (2025). Predictive Factors for Super Responder Status and Long-Term Effectiveness of Guselkumab in Psoriasis: A Multicenter Retrospective Study. Dermatol. Ther..

[15] Eyerich K., Asadullah K., Pinter A., Weisenseel P., Reich K., Paul C., Sabat R., Wolk K., Eyerich S., Lauffer F. (2024). Noninferiority of 16-Week vs 8-Week Guselkumab Dosing in Super Responders for Maintaining Control of Psoriasis: The GUIDE Randomized Clinical Trial. JAMA Dermatol..

[16] van Riel C.A.M., Boehncke W.H., Lambert J.L.W., Spuls P.I., van der Schoot L.S., van Ee I., de Jong E.M.G.J., van den Reek J.M.P.A., on behalf of the eDelphi Consortium (2026). International consensus on dose reduction of biologics for patients with psoriasis: The DR. Delphi study. J. Eur. Acad. Dermatol. Venereol..

[17] Mufti A., Maliyar K., Walton L., Vender R., Yeung J. (2020). Guselkumab dosing interval optimization in adult patients with psoriasis: A retrospective, multicenter case series. J. Am. Acad. Dermatol..

[18] McInnes I.B., Rahman P., Gottlieb A.B., Hsia E.C., Kollmeier A.P., Chakravarty S.D., Xu X.L., Subramanian R.A., Agarwal P., Sheng S. (2021). Efficacy and safety of guselkumab through one year in biologic-naive patients with psoriatic arthritis. Arthritis Rheumatol..

[19] Mease P.J., Baraliakos X., Chandran V., Soriano E.R., Nash P., Deodhar A., Rampakakis E., Shiff N.J., Chakravarty S.D., Shawi M. (2024). Effect of guselkumab administered every 8 weeks in patients with active psoriatic arthritis persists between consecutive doses and is durable: Post hoc analysis of a phase 3 randomised study. ACR Open Rheumatol..

[20] Yao Z., Hu C., Zhu Y., Xu Z., Randazzo B., Wasfi Y., Chen Y., Sharma A., Zhou H. (2018). Population pharmacokinetic modeling of guselkumab, a human IgG1lambda monoclonal antibody targeting IL-23, in patients with moderate to severe plaque psoriasis. J. Clin. Pharmacol..

[21] Lebwohl M., Langley R.G., Zhu Y., Zhou H., Song M., Shen Y.K., Lafferty K.P., Reich K. (2019). Use of dose-exposure-response relationships in phase 2 and phase 3 guselkumab studies to optimize dose selection in psoriasis. J. Eur. Acad. Dermatol. Venereol..

[22] Mehta H., Mashiko S., Angsana J., Rubio M., Hsieh Y.-C.M., Maari C., Reich K., Blauvelt A., Bissonnette R., Muñoz-Elías E.J. (2021). Differential changes in inflammatory mononuclear phagocyte and T-cell profiles within psoriatic skin during treatment with guselkumab versus secukinumab. J. Investig. Dermatol..

[23] Gossec L., Kerschbaumer A., Ferreira R.J.O., Aletaha D., Baraliakos X., Bertheussen H., Boehncke W.-H., Esbensen B.A., McInnes I.B., McGonagle D. (2024). EULAR recommendations for the management of psoriatic arthritis with pharmacological therapies: 2023 update. Ann. Rheum. Dis..

